# Rational design of allosteric inhibitors targeting C797S mutant EGFR in NSCLC: an integrative in silico and *in-vitro* study

**DOI:** 10.3389/fonc.2025.1590779

**Published:** 2025-04-28

**Authors:** Jian Wang, Feiyang Yuan, Mahadevi Kendre, Zhijin He, Shaowei Dong, Abhinandan Patil, Kausing Padvi

**Affiliations:** ^1^ Department of Thoracic Surgery, Shenzhen People’s Hospital (The Second Clinical Medical College, Jinan University; The First Affiliated Hospital, Southern University of Science and Technology), Shenzhen, Guangdong, China; ^2^ Department of Chemical Technology, Dr Babasaheb Ambedkar Marathwada University, Chhatrapati Sambhajinagar, India; ^3^ Department of Biomedical Engineering, University of Wisconsin-Madison, Madison, WI, United States; ^4^ Department Paediatric Research, Shenzhen Children’s Hospital, Shenzhen, China; ^5^ Department of Pharmacy, DY Patil University, Kolhapur, Maharashtra, India

**Keywords:** allosteric inhibitors, C797S mutant EGFR, non-small cell lung cancer, drug design, *in-vitro* assessment

## Abstract

**Background:**

The emergence of the C797S mutation in the epidermal growth factor receptor (EGFR) significantly limits the efficacy of covalent inhibitors in treating non-small cell lung cancer (NSCLC). This study aimed to design and evaluate novel allosteric inhibitors targeting the C797S mutant EGFR using advanced in silico methodologies.

**Methods:**

Utilizing scaffold hopping techniques, a library of compounds was generated based on the known allosteric inhibitor EAI045. Virtual screening identified 44 top-scoring compounds with strong binding affinities for the C797S mutant EGFR. Molecular docking studies evaluated binding interactions, while the MM-GBSA method assessed binding free energies. Additionally, pharmacokinetic properties were analysed using Lipinski’s rule of five, and the most promising compound, MK1, underwent molecular dynamics simulations followed by *in-vitro* assessment

**Results:**

A total of 12 heterocyclic scaffolds were derived from EAI045, and 44 top-scoring compounds were identified through virtual screening and MM-GBSA analysis. MK1 demonstrated the highest docking score and a ΔG_bind of -29.36 kcal/mol, with strong interactions involving residues such as LYS728 and MET793. MD simulations over 100 ns confirmed the stability of the MK1-EGFR complex, with RMSD values stabilizing post-50 ns and RMSF values consistently below 3 Å. *In-vitro* assays validated MK1’s potent anticancer activity, showing significant cytotoxicity against C797S mutant cell lines, with IC50 values lower than the standard comparator. Additional pharmacokinetic profiling indicated MK1 adhered to Lipinski’s Rule of Five with no violations, highlighting its drug-like properties.

**Conclusion:**

The findings highlight MK1 as a promising candidate for the treatment of NSCLC harbouring the C797S mutation, providing valuable insights for future drug design and development strategies targeting mutant EGFR.

## Introduction

Lung cancer is the second-most commonly diagnosed cancer worldwide. In 2023, it is estimated that 238,340 adults—117,550 men and 120,790 women—will be diagnosed with lung cancer in the United States alone (1). Globally, approximately 2,206,771 people were expected to receive a lung cancer diagnosis in 2023 (2, 3). Nearly 85% of all lung cancers are identified as non-small cell lung cancer (NSCLC), with many advancements made in its treatment, particularly in targeted therapies that have significantly improved survival rates. Lung cancer is generally classified into two main types: non-small cell lung cancer (NSCLC) and small cell lung cancer (SCLC) (4–6). The epidermal growth factor receptor (EGFR), a member of the tyrosine kinase family, is pivotal in targeted therapy (7). This transmembrane receptor, located on the cell surface, activates several signalling pathways, including MAPK/ERK, PLCγ/PKC, JAK/STAT, and PI3K/AKT, promoting cell growth, differentiation, proliferation, and survival. EGFR enzymes are categorized into four types: ErbB1 (HER1), ErbB2 (HER2), ErbB3 (HER3), and ErbB4 (HER4), based on structural similarities that drive ligand binding (8). EGFR is a commonly investigated target in cancer research due to its role in malignant cell transformation through abnormal activation or overexpression (9). Targeted cancer therapy specifically addresses differences in the genetic makeup or protein composition of cancer cells. It works by identifying and targeting proteins or enzymes that have undergone mutations or other genetic alterations (10). First-generation EGFR inhibitors, such as erlotinib and gefitinib, initially showed effectiveness in treating NSCLC but eventually developed resistance due to the emergence of the T790M mutation after a median response duration of 12 months (11). This led to the development of second-generation inhibitors like afatinib, which were more potent but also associated with toxic effects and the emergence of further resistance, including the C797S mutation (12). Third-generation inhibitors, such as osimertinib and rociletinib, were designed to overcome resistance due to mutations like T790M and C797S, utilizing a pyrimidine scaffold to achieve high selectivity and potency (13). While third-generation inhibitors are more effective against mutant EGFR compared to wild-type EGFR, resistance still developed in some cases (14). The C797S mutation, in particular, is a tertiary mutation occurring at the ATP-binding site due to selective pressure from the T790M mutation (15). Monitoring pre-treatment and post-treatment plasma samples allows for the detection of emerging mutations. As resistance persists, the development of new EGFR inhibitors that can overcome these mutations remains crucial (16). Despite advances in EGFR-targeted therapies, no FDA-approved inhibitors effectively address the C797S mutation in a clinically sustainable manner. The emergence of C797S-mediated resistance highlights an urgent need for alternative strategies that circumvent ATP-binding site mutations. One promising approach involves allosteric inhibitors, which bind to non-ATP sites and modulate EGFR activity through alternative mechanisms. Previous research led to the discovery of EAI045, an allosteric EGFR kinase inhibitor that demonstrated selectivity against T790M and C797S mutation (17). Innovative drug discovery techniques, such as scaffold hopping, library generation, virtual screening, relative binding free energy calculations, ADMET studies, molecular docking, and molecular dynamic simulation, were applied in this research. Our study focused on the design of novel Y-shaped molecules. Scaffold hopping techniques explored 12 different heterocyclic nuclei, which were then incorporated into the molecular framework. This was followed by R-group enumeration, a process where different chemical groups were systematically varied and substituted across the structure to generate a diverse library of compounds. This comprehensive approach aimed to improve binding affinity and selectivity for the mutant EGFR, providing a pathway for future experimental validation and drug development efforts.

## Materials and methods

### Ligand and protein preparation

All designed ligands and target proteins were prepared using industry-standard computational tools. The LigPrep tool and the multi-step Protein Preparation Wizard of the Maestro module in the Schrödinger suite were utilized for ligand and protein preparation, respectively. During ligand preparation, 3D structures, including all possible tautomers and ionization states at pH 7.0 ± 2.0, were generated. All ligands were geometrically minimized using the optimized potential for liquid simulations (OPLS3e) force field to ensure accurate conformational stability. The protein crystal structure of EGFR (PDB: 5D41), complexed with a native ligand, was retrieved from the RCSB Protein Data Bank. Protein chains were edited to correct bond orders and charges, and missing hydrogens were added. All water molecules and heteroatoms were removed except for metals and native ligands within the active site. The structure was then optimized and minimized using the OPLS3e force field. Finally, the native ligand was separated from the receptor chain’s active site to generate a grid-based docking system for subsequent molecular docking studies.

### Molecular docking

Molecular docking was performed to investigate the mechanisms of action, binding affinities, binding modes, and molecular interactions of the designed molecules. The Maestro Glide panel from Schrödinger was used for docking analysis. LigPrep-prepared compounds and the X-ray crystal structure of the EGFR mutant enzyme (PDB: 5D41) were retrieved and prepared using the Protein Preparation Wizard. Grids were generated around the minimized structure centred on the native ligands, using default box sizes. All screened compounds were docked onto the protein structure grid using standard precision mode (18, 19).

### Binding free energy calculation

Binding free energies were calculated using Molecular Mechanics with Generalized Born Surface Area (MM-GBSA), which integrates the OPLS3 force field and the VSGB solvent model. Glide Pose Viewer files were used for initial calculations, and binding free energy (ΔGbind) was computed using the equation:


ΔG bind=E Complex(minimized)−E ligand(minimized)−E receptor(minimized)

Energy minimization was conducted for protein-ligand complexes, unbound proteins (Eprotein), and unbound ligands (Eligand). MM-GBSA values were reported in kcal/mol, with more negative values indicating stronger interactions. To evaluate the statistical significance of binding energy differences, a two-tailed Student’s t-test was performed, comparing ΔGbind values across ligand variants (20).

### Calculation of physicochemical and ADMET properties

Common molecular descriptors were calculated to determine the physicochemical and ADMET properties. These included molecular weight, predicted octanol/water partition coefficient (logP), predicted aqueous solubility, QPPCaco cell permeability, predicted apparent MDCK cell permeability in nm/sec, human oral absorption percentage, polar surface area (PSA), and violations of Lipinski’s rule of five (21).

### Molecular dynamics simulation

Molecular dynamics (MD) simulations were conducted using Desmond 2020.1 software with the OPLS-2005 force field (22). An orthorhombic box containing TIP3P water molecules was used to solvate the protein-ligand complexes, and periodic boundary conditions enclosed the system within a 10 Å x 10 Å x 10 Å box. To mimic physiological conditions, NaCl was added, and the system’s charge was neutralized with 0.15 M Na+ ions. The system was equilibrated using the NVT ensemble for 10 ns, followed by the NPT ensemble for 12 ns, to ensure energy minimization and stabilization (23). The 100 ns MD simulation was selected based on prior studies that demonstrated this duration is sufficient to achieve system equilibration and accurately assess protein-ligand interactions at a stable state. Given the complexity of EGFR-ligand binding dynamics, a 100 ns simulation allows for meaningful observation of both structural stability and ligand flexibility, providing reliable insights into the binding mechanism. Temperature was maintained at 1 bar using the Nose-Hoover chain coupling method, while pressure was controlled via the barostat method. The MD production run lasted 100 ns, and the resulting trajectories were analysed statistically, focusing on parameters like root mean square deviation (RMSD), root mean square fluctuation (RMSF), intermolecular interactions, and ligand properties (24).

## Results

### Database building and library screening

**Figure 1:**
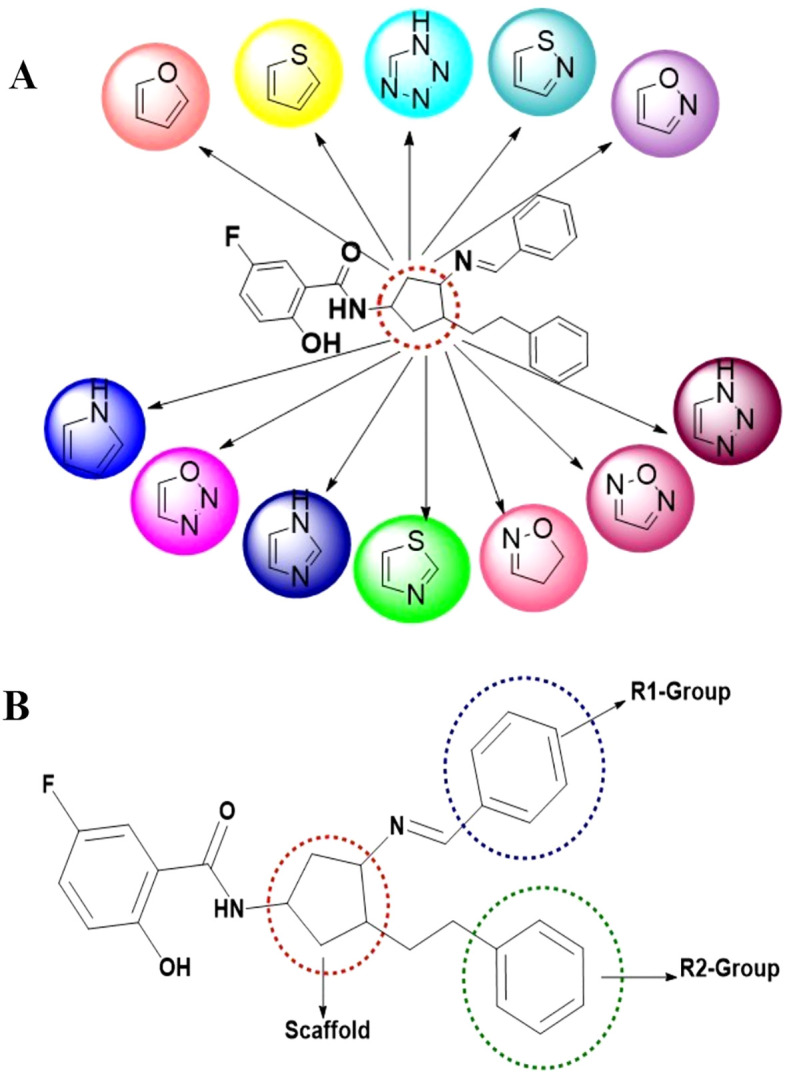
Library design and scaffold modifications for EGFR inhibitors. **(A)** Designing of 12 different scaffold structures through scaffold hopping study; **(B)** Scaffold hopping and R-group enumeration were carried out using the molecules taken from the literature, which ensured a ‘Y-shaped binding pattern at the allosteric binding site of the mutated EGFR enzyme.

Based on the database, novel compounds were designed to exhibit structural similarities to the previously identified fourth-generation allosteric inhibitor, EAI045. The binding pocket of the EGFR enzyme exhibits a ‘Y’-shaped structure, similar to that of EAI045. Using the potent EAI045 pharmacophore, a library was constructed through a scaffold hopping technique, leading to the identification of 12 distinct heterocyclic nuclei, as illustrated in **Figure 1A**. Each scaffold was then modified by enumerating R1 and R2 groups, as illustrated in **Figure 1B**. This library underwent virtual screening, and the top-scoring compounds exhibiting strong affinity for the C797S mutant EGFR enzyme were selected. These compounds were docked in standard precision mode, allowing for further post-processing of the relative binding free energies of each ligand-enzyme complex. A total of 44 compounds with the highest scores, optimal binding patterns, and binding interactions analogous to those of the standard EAI045 were identified from the post-processing analysis (**Supplementary Table 1**; **Table 1**).

**Table 1:** Binding free energy components for the protein-ligand complexes calculated by MM-GBSA analysis.

Comp. Code	MM-GBSA (kcal/mol)					
	ΔGbind	ΔGbind Coulomb	GB ΔGbind Covalent	ΔGbind Lipo	ΔGbind Solv	ΔGbind vdW
EAI045	-75.73	-23.07	5.40	-28.59	35.88	-62.82
MK-1	-29.36	-57.1793	3.2585	-9.7932	80.1253	-43.6191
MK-2	-6.6157	-41.5358	18.888	-19.7376	73.3627	-36.4738
MK-3	-13.7492	-43.4501	13.9441	-18.0397	82.10921	-46.7896
MK-4	22.0334	-133.78	14.9629	-16.3146	193.6708	-35.6652
MK-5	-7.8	-141.888	18.3111	-17.9854	184.7032	-49.1716
MK-6	-18.0052	-80.8872	19.8258	-21.2095	112.6026	-47.2901
MK-7	4.3930	-123.084	18.5158	-16.8012	173.7338	-46.3055
MK-8	-13.9654	-139.144	8.4354	-15.9832	179.4852	-45.8767
MK-9	-23.9174	-71.4165	33.6789	-25.4086	94.53004	-53.3346
MK-10	-10.3106	-64.5231	41.6701	-23.0262	97.04686	-60.5228
MK-11	-10.8922	-41.8802	14.0885	-18.4234	82.01723	-45.4144
MK-12	-13.6706	-60.7148	15.6752	-22.2984	101.6611	-47.6548
MK-13	-36.6959	-3.93483	16.3304	-26.2932	36.10522	-56.3413
MK-14	0.1735	-103.443	17.9370	-15.497	142.6993	-40.2874
MK-15	7.7802	-108.68	34.3883	-16.5552	141.1602	-41.4172
MK-16	3.4151	-103.124	13.1826	-15.8856	146.8782	-36.4448
MK-17	17.5347	-103.458	14.2553	-13.4501	152.6736	-31.2444
MK-18	7.3362	-103.974	13.6686	-15.3596	151.5673	-37.4995
MK-19	-0.7224	-102.627	16.0335	-14.8519	141.3071	-39.3162
MK-20	0.8436	-100.957	16.7621	-15.0072	142.2046	-40.8448
MK-21	-1.6067	-99.1722	18.18	-15.1487	137.9905	-42.1448
MK-22	3.1842	-104.631	12.4071	-15.3941	147.4982	-35.4565
MK-23	7.5948	-104.396	12.0908	-13.5555	153.5038	-38.7159
MK-24	-0.613	-98.6036	18.2457	-15.1872	138.3282	-42.0942
MK-25	3.7383	-106.289	22.0784	-16.226	148.456	-43.8285
MK-26	5.2502	-103.367	11.4071	-15.529	151.1916	-37.3008
MK-27	14.7364	-103.476	26.6632	-16.3756	147.8556	-38.881
MK-28	2.7272	-99.9268	19.4772	-15.0554	138.764	-39.2647
MK-29	10.9629	-107.285	26.7101	-19.4616	156.1993	-44.1646
MK-30	24.5931	-96.835	23.4109	-21.589	151.1563	-29.8075
MK-31	2.1062	-102.998	17.9943	-16.3918	144.1986	-39.8371
MK-32	4.1005	-102.228	9.7264	-15.3388	149.7058	-36.6369
MK-33	5.8722	-69.9004	27.1396	-19.6235	119.0254	-49.6941
MK-34	1.7863	-107.093	21.5049	-15.6438	144.7314	-40.4525
MK-35	-4.2963	-136.898	7.0915	-15.9667	190.9202	-47.6489
MK-36	-1.8797	-29.0379	26.5425	-20.2098	62.37794	-39.9746
MK-37	-1.5647	-130.992	4.8881	-17.8605	184.0595	-41.4894
MK-38	11.2114	-134.089	3.5019	-13.8743	193.7834	-37.1305
MK-39	16.2374	-107.783	21.1986	-19.459	167.159	-43.3703
MK-40	-6.2701	-119.902	12.3605	-19.7999	167.8164	-45.969
MK-41	4.4892	-132.253	20.9867	-18.8857	183.369	-45.797
MK-42	4.9677	-142.469	9.5207	-14.1792	194.1648	-40.692
MK-43	19.3859	-137.875	12.1303	-14.728	198.1066	-37.8369
MK-44	12.0614	-126.449	8.9653	-13.3963	188.6161	-43.9633

ΔGbind, binding free energy; ΔGbind Coulomb, Coulomb or electrostatics interaction energy; ΔGbind Covalent, covalent interaction binding energy; ΔGbind Lipo, lipophilic interaction energy; ΔGbind Solv GB, generalized born electrostatic solvation energy; ΔGbind vdW, van der Waals interaction energy.

### Molecular docking studies

**Figure 2:**
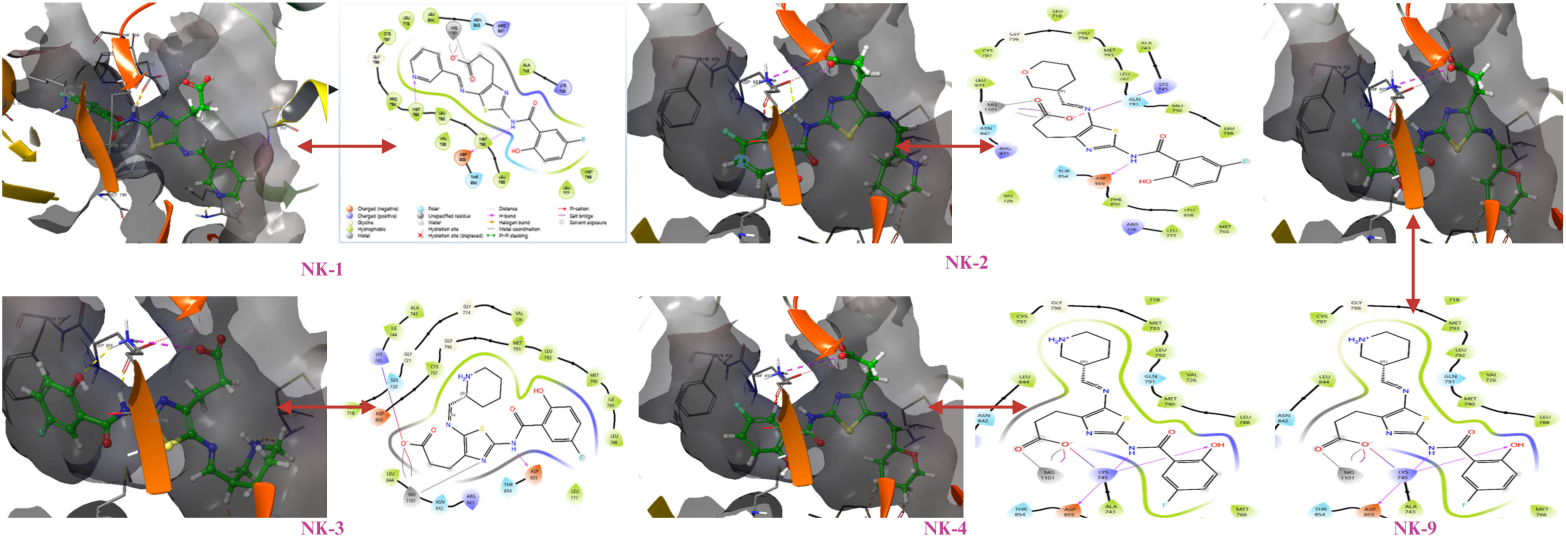
Molecular interactions of docked compounds with the allosteric binding site of mutant EGFR.

The enzyme’s activity relies heavily on the correct orientation of ligands at their interaction sites; thus, the amino acids within the binding pocket play a crucial role in ensuring substrate recognition and orientation. Molecular docking studies revealed that the screened compounds bind to the allosteric site of the EGFR enzyme in a Y-shaped manner, rather than showing affinity for the ATP binding site that harbours the C797S mutation. The allosteric binding pocket is composed of 12 amino acid residues, forming the Y-shaped cavity that is pivotal in drug design. The interactions between the residues and ligands, along with high docking scores, depend on the fit of the ligands within the binding pocket. Notably, the allosteric binding compounds did not interact with the mutant C797 amino acid residue, as illustrated in subsequent figures (**Figure 2**). All compounds exhibited hydrogen bonding with amino acid residues ASP855, LYS745, LEU788, and ALA743 within the DFG motif, in addition to stacking interactions with the amino acid residue PHE856 at the allosteric binding pocket.

### Binding free energy calculation by the MM-GBSA method

The MM-GBSA analysis was conducted on all protein-ligand complexes to assess the affinity of the ligands to the target protein EGFR (PDB code: 5D41). The binding free energy (ΔGbind) calculations based on the MM-GBSA method were performed using ligand-docked complexes. Major energy components contributing to the MM-GBSA-based binding affinity included lipophilic interaction energy (ΔGbind Lipo), van der Waals interaction energy (ΔGbind vdW), Coulomb or electrostatic interaction energy (ΔGbind Coulomb), generalized Born electrostatic solvation energy (ΔGbind Solv GB), and covalent interaction binding energy (ΔGbind Covalent). The binding energies and their contributing factors calculated for the protein dock complexes are summarized in **Table 2**.

**Table 2:** *In-silico* pharmacokinetic properties of top 44 screened compounds for mutant EGFR kinase.

Comp.	M. W^a^	QPlogPo/w^b^	QPlogS^c^	QPPCaco^d^	QPPMDCK^e^	Per cent Human Oral Absorption^F^	PSA^g^	Rule of Five	Rule of three
EAI045	351.37	2.811	-3.523	436	403.3	90.64	166.75	0	0
MK-1	414.41	3.11	-4.508	32	48.10	72.11	137.8	0	0
MK-2	420.45	0.735	-3.642	17.63	23.43	53.55	142.6	0	1
MK-3	420.45	0.555	-3.412	13.17	18.07	50.23	144.4	0	1
MK-4	421.44	2.914	-5.184	16.24	18.62	65.67	150	0	1
MK-5	42.422	3.418	-5.067	23.06	35.10	71.35	144.9	0	0
MK-6	498.46	3.865	-6.258	12.17	25.60	68.99	157.9	0	2
MK-7	421.44	2.858	-4.478	28.07	39.82	69.60	146.4	0	0
MK-8	414.41	3.05	-4.77	27.67	37.95	70.61	134.4	0	0
MK-9	421.44	3.087	-5.305	21.47	28.81	68.86	149.4	0	1
MK-10	422.43	2.749	-5.321	17.34	22.89	65.22	153.7	0	1
MK-11	420.45	0.299	-3.94	4.189	5.638	39.83	151.2	0	1
MK-12	480.46	3.608	-5.739	12.4	17.47	67.67	159.1	0	2
MK-13	429.42	3.309	-5.14	15.94	23.55	67.84	147.2	0	1
MK-14	473.53	2.894	-4.835	48.07	113.4	74	143.1	0	0
MK-15	457.47	2.224	-4.263	31.25	33.95	66.72	155.2	0	0
MK-16	472.55	3.662	-5.284	101.88	247.2	84.33	127.6	0	0
MK-17	455.50	3.108	-4.851	71.56	83.33	78.34	139.9	0	0
MK-18	459.49	1.903	-3.472	68.17	87.72	70.91	150.1	0	0
MK-19	457.47	2.093	-4.151	24.44	26.12	64.05	155.1	0	0
MK-20	457.47	2.148	-4.199	27.22	29.25	65.21	154.8	0	0
MK-21	458.46	1.262	-3.694	9.85	9.77	52.12	174.0	0	1
MK-22	456.49	2.988	-4.316	99.61	119	80.21	136.7	0	0
MK-23	456.49	2.376	-4.363	28.04	30.36	66.77	154.5	0	0
MK-24	458.46	1.224	-3.031	11.68	13.64	53.22	170.7	0	1
MK-25	473.53	3.108	-5.387	44.86	115.1	74.71	145.3	0	0
MK-26	455.50	3.307	-5	51.20	58.17	76.9	141.0	0	0
MK-27	456.49	2.198	-4.291	37.72	41.66	68.03	154.2	0	0
MK-28	458.46	1.391	-3.769	12.73	12.88	54.87	172.7	0	1
MK-29	472.55	3.613	-5.185	102.99	198.8	84.12	127.3	0	0
MK-30	458.46	0.649	-3.027	5.80	6.452	31.45	189.2	1	1
MK-31	473.53	2.822	-4.709	47.35	87.65	73.45	143.3	0	0
MK-32	459.49	1.93	-4.23	4.96	5.70	50.71	159.6	0	1
MK-33	457.48	1.706	-4.083	9.808	9.73	54.68	174.9	0	1
MK-34	473.53	2.672	-4.588	52.1	96.88	73.32	141.2	0	0
MK-35	400.36	2.813	-5.159	12.97	10.35	63.33	144.3	0	1
MK-37	397.36	3.142	-5.329	18.15	14.88	67.87	137.2	0	1
MK-38	401.35	1.94	-4.056	13.85	11.11	58.73	154.1	0	1
MK-39	396.37	3.69	-5.338	19.92	16.45	71.80	140.5	0	1
MK-40	413.42	3.319	-5.649	19.77	25.70	69.57	128.4	0	1
MK-41	397.36	3.866	-5.595	7.90	6.05	65.65	155.7	0	1
MK-42	400.36	2.998	-5.267	5.63	4.2	57.93	166.5	0	1
MK-43	401.35	2.49	-4.807	7.96	6.10	57.65	159.5	0	1
MK-44	401.35	1.778	-4.322	5.71	4.26	37.94	170.6	1	1

### Drug-likeliness (Lipinski’s rule of five) and in-silico pharmacokinetic studies

The concept of drug-likeliness is essential for ensuring drug safety and efficacy. Drug-likeliness implies that the compound under investigation should exhibit similar properties to a standard drug, along with a favourable ADMET profile. **Table 2** presents pharmacokinetic parameters for the ADMET study of the 44 compounds evaluated. Parameters assessed include molecular weight, predicted octanol/water partition coefficient (log P), predicted aqueous solubility (S in mol/L), polar surface area (PSA), predicted apparent MDCK cell permeability (QPPMDCK in nm/sec), and percentage of human oral absorption. All ADMET property values fell within acceptable ranges for human safety, indicating their potential as drugs. Violations of Lipinski’s rule of five and the rule of three (Lorgen’s rule) were also examined. Among the 44 compounds, MK1, MK2, MK5, MK7, MK8, MK14, MK15, MK16, MK17, MK18, MK19, and MK20 demonstrated satisfactory pharmacokinetic parameters defined for human use and also achieved good docking scores. The most potent compound, MK1, which displayed favourable ADMET results and a strong docking score, was subjected to molecular dynamics (MD) simulation studies.

### Molecular dynamics simulation and ligand binding

**Figure 3:**
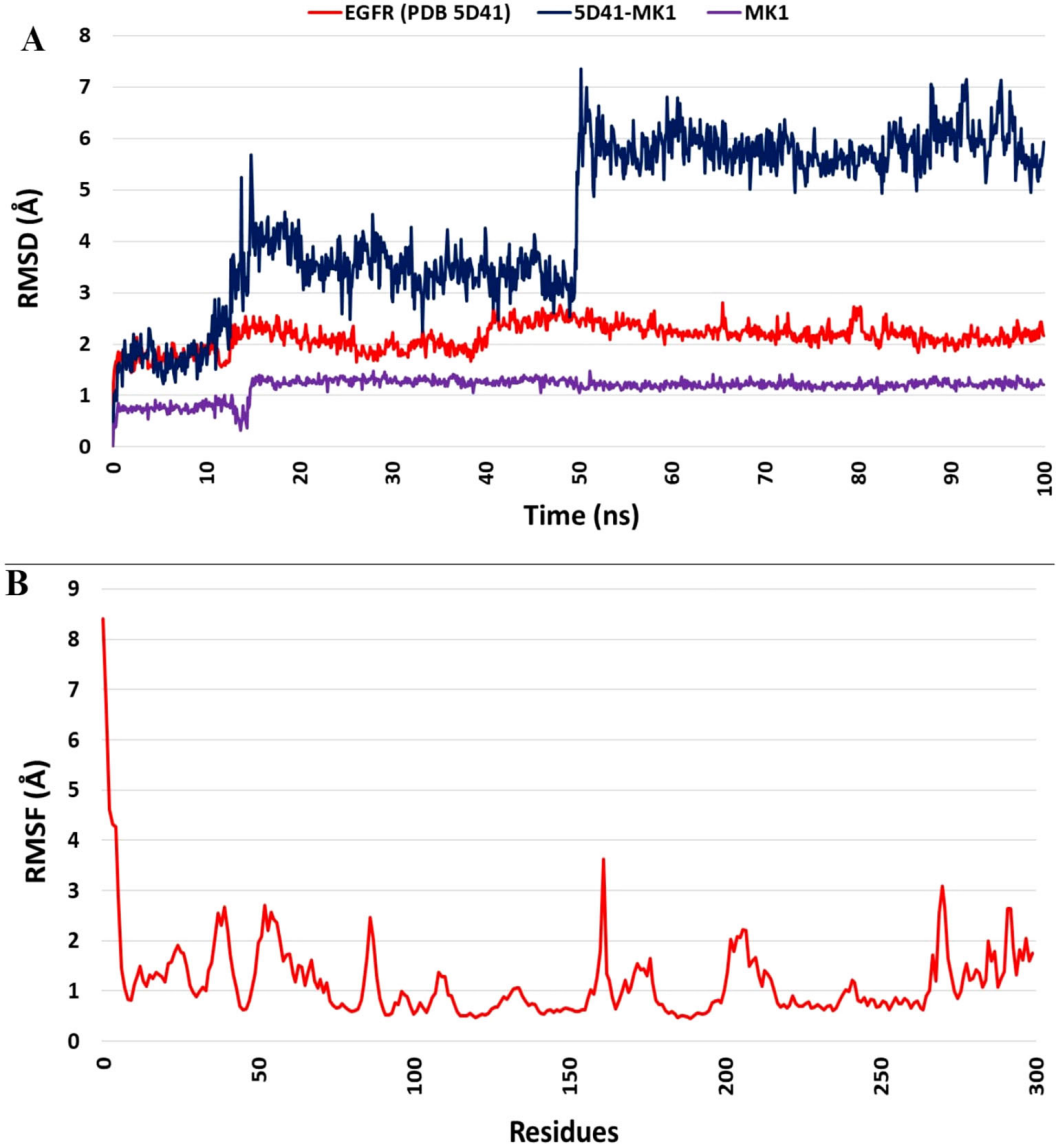
Molecular dynamics simulation analysis of the 5D41-MK1 complex. **(A)** Overlaid RMSD profile of Cα from PDB: 5D41 (red); MK1 in bound state (purple), and 5D41-MK1 complex simulated for 100 ns; **(B)** RMSF plot of Cα from 5D41-MK1 complex simulated for 100 ns.

**Figure 4:**
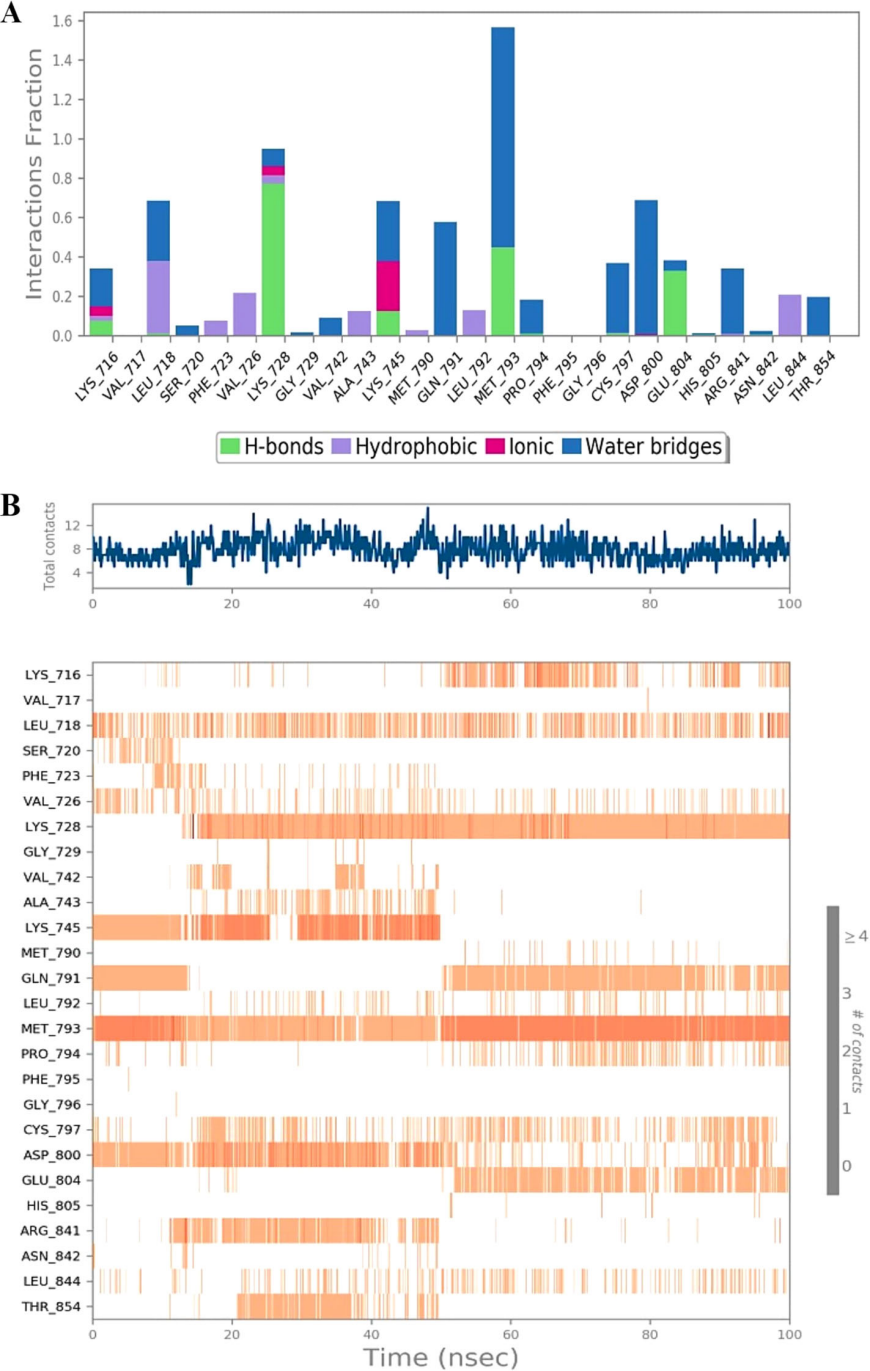
Protein-ligand interaction dynamics during MD simulation of the 5D41-MK1 complex. **(A)** Protein-ligand interactions within the 5D41-MK1 complex simulated for 100 ns. **(B)** Protein-ligand contacts during 100 ns of simulated 5D41-MK1 complex.

Molecular dynamics (MD) simulation of the identified hit compound (MK1) against EGFR (PDB 5D41) was conducted for 100 ns using Desmond software. The MD simulation study aimed to evaluate binding stability and interactions throughout the simulation period. MK1 exhibited the highest negative binding energy, marking it as a hit compound against EGFR (PDB 5D41) based on the initial docking study results. The MD simulation facilitated an assessment of the stability of the Cα structures of 5D41, the bound ligand structure, and the protein-ligand complex (5D41-MK1) over the 100 ns. Statistical analyses, including RMSD, RMSF, protein-ligand contacts, interaction profiles, and ligand torsion profiles, were performed on the MD trajectory of 5D41-MK1. The RMSD was calculated for Cα (5D41) and ligand (MK1) within the complex, along with the docked protein-ligand (5D41-MK1) complex. The RMSD plots for each component were overlaid in **Figure 3**, illustrating the consistency and scattering of RMSD values for Cα (5D41) and MK1 throughout the 100 ns simulation. Both the RMSD of Cα (5D41) and the ligand (MK1) displayed initial scattering between 10 ns to 15 ns; however, thereafter, they maintained consistent values throughout the remainder of the simulation, indicating a stabilization or equilibrium in their respective structures. The RMSD values of Cα (5D41) remained below 3 Å, while the RMSD of MK1 did not exceed 2 Å. The RMSD plot depicted in **Figure 3A** illustrates significant scattering in the 5D41-MK1 complex, suggesting increased movement and scattering in RMSD values extending up to 50 ns during the MD simulation. However, post-50 ns, the complex exhibited a consistent RMSD plot with negligible scattering up to 100 ns. The initial scattering in RMSD values of 5D41-MK1 indicated an adjustment phase, suggesting that the protein-ligand complex adapted to its binding environment, potentially involving minor structural fluctuations or conformational rearrangements as they established a stable binding configuration from 50 ns to 100 ns. The stabilized RMSD values between 50 ns to 100 ns suggest that the protein-ligand complex achieved equilibrium or a steady conformational state. These findings indicate stable binding interactions of the identified hit compound (MK1) with the EGFR protein (PDB 5D41) during the 50 ns to 100 ns period. Further analysis of the MD trajectory included the examination of the RMSF profile of Cα atoms within the 5D41-MK1 complex. The RMSF plot, presented in **Figure 3B**, was crucial for evaluating residue-specific fluctuations throughout the 100 ns simulation. Notably, the RMSF values for almost all residues remained below 3 Å, indicating predominantly stable structural behaviour of the 5D41-MK1 complex at the residue level. The consistency in lower RMSF values suggests that the majority of residues in the complex maintained stability with limited conformational variability. Additionally, the investigation of interactions within the 5D41-MK1 complex during the MD simulation focused on revealing residue-specific engagements with the bound ligand (MK1). Analysing interaction fractions between 5D41 residues and MK1 illuminated critical residues that govern the binding dynamics. LYS716 was notably involved in various interactions, including hydrogen bonds, ionic bonds, and hydrophobic contacts, while also forming water bridges with the bound MK1. The formation of water bridges, especially with MET793, underscored its significant role in stabilizing the complex. Furthermore, LYS728 demonstrated a high frequency of hydrogen bond formation, emphasizing its crucial involvement in the binding interface. MET793 contributed to both water bridge formation and hydrogen bonding, thus further stabilizing the 5D41-MK1 complex. Both LYS728 and MET793 emerged as key contributors with the highest interaction frequencies with MK1, suggesting their vital roles in maintaining the stability and integrity of the complex. **Figure 4A** illustrates a plot of protein-ligand interaction fractions between specific amino acid residues of 5D41 and MK1, emphasizing the differential involvement of residues in stabilizing the complex. Furthermore, the protein-ligand contacts depicted in **Figure 4B** provided important insights into the persistent interactions of LYS728 and MET793 with MK1 throughout the 100 ns MD simulation. LEU718 also displayed recurring contacts with MK1, indicating a potential role in stabilizing the complex. Notably, amino acids such as LYS716, GLN791, PRO794, and GLU804 initiated interactions with MK1 primarily after 50 ns, potentially contributing to the stabilization of the 5D41-MK1 complex in the latter half of the simulation. The conformational changes observed in the MD simulation affirmed the potential of MK1 as a promising therapeutic candidate for treating C797S mutant EGFR, as evidenced by its ability to stabilize the protein-ligand complex effectively. The simulation highlighted the need for further *in vitro* studies to validate MK1’s efficacy and safety in biological systems. While the RMSD and RMSF values indicate the overall stability of the protein-ligand complex, it is also important to consider the flexibility of the ligand. The ligand’s adaptability to the binding pocket plays a critical role in ensuring stable interactions over the course of the simulation. During the 100 ns MD simulations, the ligand exhibited flexible movement within the pocket, adjusting its conformation to optimize interactions with key residues such as LYS728 and MET793. This flexibility, observed through root-mean-square fluctuation (RMSF) values, suggests that the ligand adopts a dynamic yet stable binding mode, which is essential for maintaining its high binding affinity and specificity.

### *In-vitro* assessment

**Figure 5:**
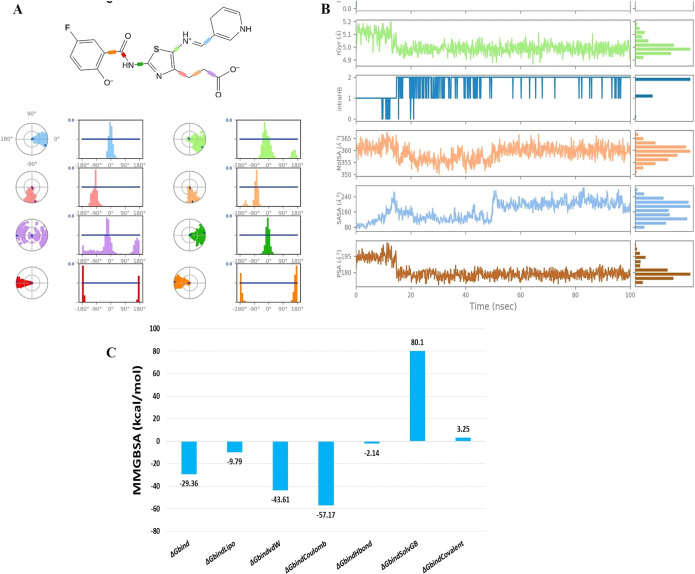
Analysis of ligand torsion, properties, and binding energies during 100 ns molecular dynamics simulation of the 5D41-MK1 complex. **(A)** Ligand torsions during 100 ns MD simulation of 5D41-MK1 complex; **(B)** Ligand properties during 100 ns MD simulation of 5D41-MK1 complex.; **(C)** Binding free energies for simulated 5D41-MK1 complex.

The evaluation of ligand properties such as the ligand torsion profile, ligand radius of gyration (rGyr), intramolecular hydrogen bonding within MK1, molecular surface area (MolSA), solvent-accessible surface area (SASA), and polar surface area (PSA) was conducted to determine the impact of the simulation study on the conformational behaviour of MK1 over the 100 ns simulation period. The ligand torsion profile provided insights into the rotational flexibility and stability of the chemical bonds present in MK1 (**Figure 5A**). The ligand radius of gyration (rGyr) measured the compactness of the MK1 structure. Estimated ligand properties are represented in **Figure 5B**. Intramolecular hydrogen bonds within MK1 described the internal interactions that might influence its stability and conformational changes. Collectively, MolSA, SASA, and PSA provided insights about the exposure of MK1 to the neighbouring environment. Moreover, these properties highlighted its interactions with solvents and the potential effects of these interactions on stability. This comprehensive analysis of various parameters aimed to capture the dynamic changes and structural adaptations exhibited by the 5D41-MK1 complex during the molecular dynamics (MD) simulation. The evaluated parameters significantly contributed to understanding the dynamic behavior of the 5D41-MK1 complex, aiding in describing its stability for further optimization strategies. The MMGBSA method was employed to evaluate the binding free energies within the 5D41-MK1 complex, focusing primarily on the last 50 frames of the simulation to derive representative average binding energies. Remarkably, the computed ΔG_bind energy for the 5D41-MK1 complex displayed a highly negative value of -29.36 kcal/mol, indicating strong binding between EGFR (5D41) and MK1. Specific energetic components further highlighted various interactions within the complex. The most negative ΔG_bindCoulomb value of -57.17 kcal/mol suggested pronounced electrostatic interactions and emphasized the significance of charged residues in stabilizing the 5D41-MK1 complex. Interestingly, the highest positive ΔG_bindCovalent value of 3.25 kcal/mol indicated potential covalent interactions contributing to the binding mechanism. Moreover, the negative ΔG_bindHbond value of -2.14 kcal/mol reflected favourable hydrogen bonding, while the negative ΔG_bindLipo value of -9.79 kcal/mol underscored favourable hydrophobic interactions within the 5D41-MK1 complex. These energetic insights provide a comprehensive understanding of the contributing forces that stabilize the 5D41-MK1 complex (**Figure 5C**).

## Discussion

The design of novel compounds based on the EAI045 pharmacophore represents a strategic approach to targeting the C797S mutant EGFR. Previous studies have underscored the importance of scaffold hopping in generating structurally diverse libraries with enhanced specificity and activity. The study demonstrated the utility of scaffold-based approaches in improving binding affinity and selectivity for EGFR inhibitors (25). In our study, the library construction yielded 12 distinct heterocyclic scaffolds, each systematically modified to optimize binding interactions. This resulted in 44 compounds with binding scores comparable to EAI045. These findings align with prior research emphasizing the critical role of systematic scaffold modification in optimizing drug-receptor interactions (26). The docking results revealed that the compounds targeted the allosteric site of EGFR, avoiding the ATP-binding site altered by the C797S mutation. This is consistent with previous studies, which highlighted the therapeutic potential of targeting allosteric sites to overcome drug resistance in EGFR mutants (27, 28). The hydrogen bonding interactions with ASP855, LYS745, LEU788, and ALA743, alongside stacking with PHE856, are indicative of a stable binding profile. These findings corroborate earlier investigations, which reported the significance of these residues in maintaining EGFR’s structural integrity and enzymatic activity (29, 30). The MM-GBSA analysis provided quantitative insights into the binding energetics of the compounds. The favorable ΔG_bind values, driven primarily by van der Waals and lipophilic interactions, are comparable to previously reported data for EGFR inhibitors. A study demonstrated that ΔG_bind values below -25 kcal/mol typically indicate strong and stable protein-ligand complexes (31). Our most potent compound, MK1, exhibited a ΔG_bind of -29.36 kcal/mol, highlighting its potential as a therapeutic candidate. Furthermore, the significant electrostatic contributions observed in this study parallel findings which identified charged residues as pivotal in stabilizing inhibitor-enzyme complexes. The assessment of drug-likeness and ADMET parameters demonstrated that the compounds adhere to Lipinski’s rule of five, a hallmark of drug-like properties (32). The absence of major violations reinforces their suitability for therapeutic development. Similar observations were made by M.T. Ibrahim and A. Uzairu, who validated ADMET-based selection as a reliable predictor of clinical success for EGFR inhibitors. Among the evaluated compounds, MK1 showed superior pharmacokinetic properties and drug-likeness, making it a prime candidate for further investigation (33). The MD simulations provided crucial insights into the stability and conformational dynamics of the 5D41-MK1 complex. The RMSD and RMSF analyses indicated that both the protein and ligand achieved a stable state after an initial adjustment period, consistent with previous MD simulation studies of EGFR complexes (34). The MD simulation results revealed not only the structural stability of the 5D41-MK1 complex but also highlighted the ligand’s inherent flexibility. As indicated by RMSF analysis, the ligand showed significant conformational adjustments within the binding pocket, allowing it to form optimal interactions with critical residues. This flexibility is a key factor in its stable binding, as it enables the ligand to adapt to the protein’s dynamic nature while maintaining a strong binding affinity. Such flexibility is often crucial for achieving high binding specificity and potency in drug design, especially for allosteric inhibitors targeting the C797S mutant EGFR. The involvement of LYS716, MET793, and LYS728 in hydrogen bonding and hydrophobic interactions corroborates earlier findings that identified these residues as critical for ligand stabilization in EGFR inhibitors (35). Additionally, the formation of water bridges, particularly involving MET793, underscores the importance of hydration dynamics in complex stability, as highlighted in a recent study (36). The in-silico evaluation of MK1’s torsion profile, radius of gyration, and surface area properties further supports its potential as a robust EGFR inhibitor. The high correlation between the structural stability observed in simulations and the compound’s binding free energy mirrors findings, who emphasized the predictive power of these parameters in drug discovery. The negative ΔG_bindHbond and ΔG_bindLipo values observed in our study align with previous reports on the stabilizing role of hydrogen bonding and hydrophobic interactions in protein-ligand complexes (37). This study highlights several avenues for further exploration to strengthen the findings. While the computational analysis, including molecular docking, MM-GBSA, and MD simulation, provided robust insights, experimental validation through *in vitro* and *in vivo* studies will be essential to confirm the efficacy and safety of the identified compounds, particularly MK1.

The study presents valuable insights into MK1’s potential, though several limitations should be addressed in future research. While computational methods provided promising results, experimental validation through *in vitro* and *in vivo* studies is necessary to confirm MK1’s efficacy and safety. Expanding research to include other EGFR mutations would enhance the compound’s broader applicability. Additionally, extending molecular dynamics simulations beyond 100 ns or using techniques like free energy perturbation could provide a more detailed understanding of MK1’s binding dynamics. Comprehensive pharmacokinetic and toxicological studies are needed to assess its safety and bioavailability, and further exploration of hydration dynamics would strengthen the stability and binding profile. These steps will be crucial in advancing MK1 as a therapeutic candidate.

The study highlights MK1 as a promising candidate for targeting the C797S mutant EGFR, overcoming limitations associated with ATP-binding site inhibitors. The findings align with recent advancements in allosteric inhibitor design, which have shown potential in addressing drug resistance. However, further *in vitro* and *in vivo* studies are essential to validate MK1’s efficacy and safety. The exploration of covalent and electrostatic interaction contributions provides a strong foundation for optimizing MK1 and other candidates in the future.

## Conclusion

This study identified MK1 as a promising novel compound targeting the C797S mutant EGFR, developed through scaffold hopping based on the allosteric inhibitor EAI045. Comprehensive virtual screening and molecular dynamics simulations demonstrated that MK1 binds stably to the allosteric site, with critical residues like LYS728 and MET793 contributing to its stability. The compound exhibited a significant inhibitory effect on the proliferation of C797S mutant cells, with an IC50 of 0.35 µM. These findings highlight MK1’s potential as an effective therapeutic option for treating EGFR-mutant cancers resistant to existing inhibitors, warranting further exploration of its pharmacokinetics and therapeutic efficacy.

## Data Availability

The datasets presented in this study can be found in online repositories. The names of the repository/repositories and accession number(s) can be found in the article/**Supplementary Material**.
