# Supplementary material for: Rational design of allosteric inhibitors targeting C797S mutant EGFR in NSCLC: an integrative in silico and in-vitro study
Source: Front Oncol. 2025 Apr 28;15:1590779. doi: 10.3389/fonc.2025.1590779 (PMC12066255; doi:10.3389/fonc.2025.1590779)
Supplement: Supplementary file 1 [file Table1.docx]

**Supplementary Table-1. Comparison of docking score, glide score, and binding energy along with interacting residues of the top 44 hits and standard EAI045.**

| Sr. No. | Structure | Docking Score | G- Score | Binding Energy | Residue |
| --- | --- | --- | --- | --- | --- |
|  |   EAI045 | -10.599 | -10.603 | -107.531 | ASP855  LYS745  PHE856 |
|  |   MK-1 | -13.348 | -13.519 | -91.446 | ASP855  MET793  MG1101 |
|  |   MK-2 | -12.080 | -12.684 | -69.645 | ASP855  LYS745  MG1101 |
|  |   MK-3 | -12.059 | -12.641 | -84.463 | ASP855  LYS745  MG1101 |
|  |   MK-4 | -11.594 | -12.176 | -88.295 | ASP855  LYS745  MG1101  LEU788  ALA743 |
|  |   MK-5 | -11.574 | -11.767 | -87.051 | - |
|  |   MK-6 | -11.572 | -12.814 | -83.147 | - |
|  |   MK-7 | -11.487 | -12.069 | -83.201 | ASP855  LYS745  MG1101  LEU788  ALA743  MET793 |
|  |   MK-8 | -11.231 | -11.402 | -86.128 | ASP855  LYS745  MG1101 |
|  |   MK-9 | -11.212 | -11.794 | -75.724 | - |
|  |   MK-10 | -10.760 | -11.504 | -75.060 | ASP855  LYS745  MG1101 |
|  |   MK-11 | -10.694 | -11.276 | -70.212 | - |
|  |   MK-12 | -10.496 | -11.657 | -74.142 | - |
|  |   MK-13 | -8.409 | -9.229 | -75.814 | - |
|  |   MK-14 | -9.833 | -11.085 | -120.729 | ASP855  MET793  MG1101 |
|  |   MK-15 | -9.650 | -10.902 | -108.046 | ASP855  MET793  MG1101 |
|  |   MK-16 | -9.605 | -10.857 | -111.054 | ASP855  MET793  MG1101 |
|  |   MK-17 | -9.422 | -10.674 | -98.495 | ASP855  MET793  MG1101 |
|  |   MK-18 | -9.394 | -10.646 | -100.651 | - |
|  |   MK-19 | -9.249 | -10.501 | -117.200 | - |
|  |   MK-20 | -9.169 | -10.422 | -114.858 | - |
|  |   MK-21 | -9.165 | -10.417 | -114.415 | - |
|  |   MK-22 | -9.057 | -10.309 | -112.568 | - |
|  |   MK-23 | -9.057 | -10.343 | -110.157 | LYS745  MET793  MG1101 |
|  |   MK-24 | -9.055 | -10.307 | -114.152 | - |
|  |   MK-25 | -9.051 | -10.303 | -104.465 | - |
|  |   MK-26 | -8.833 | -10.085 | -108.214 | - |
|  |   MK-27 | -8.726 | -9.978 | -71.918 | - |
|  |   MK-28 | -8.682 | -9.934 | -90.122 | - |
|  |   MK-29 | -8.670 | -9.922 | -112.301 | - |
|  |   MK-30 | -8.654 | -9.258 | -88.072 | - |
|  |   MK-31 | -8.632 | -9.884 | -96.434 | - |
|  |   MK-32 | -8.608 | -9.860 | -108.506 | - |
|  |   MK-33 | -8.551 | -9.276 | -118.667 | - |
|  |   MK-34 | -8.454 | -9.706 | -102.487 | - |
|  |   MK-35 | -10.134 | -11.357 | -138.842 | - |
|  |   MK-36 | -10.026 | -11.239 | -112.384 | LYS745  MET793  MG1101 |
|  |   MK-37 | -9.901 | -11.091 | -146.717 | LYS745  MG1101 |
|  |   MK-38 | -9.887 | -10.951 | -130.846 | - |
|  |   MK-39 | -9.491 | -10.658 | -114.995 | - |
|  |   MK-40 | -9.314 | -10.470 | -123.565 | - |
|  |   MK-41 | -9.282 | -11.172 | -104.883 | - |
|  |   MK-42 | -8.788 | -10.650 | -132.605 | - |
|  |   MK-43 | -8.775 | -10.578 | -129.381 | - |
|  |   MK-44 | -8.246 | -10.011 | -128.931 | - |
